# Nutrition and Vascular Supply of Retinal Ganglion Cells during Human Development

**DOI:** 10.3389/fneur.2016.00049

**Published:** 2016-04-07

**Authors:** Paul Rutkowski, Christian Albrecht May

**Affiliations:** ^1^Ophthalmologist, 282 Harrison Ave., Harrison, NY, USA; ^2^Department of Anatomy, Medical Faculty Carl Gustav Carus, Technische Universität Dresden, Dresden, Germany

**Keywords:** retinal ganglion cells, oxygen supply, fetal, adult, normal development, retinal capillaries, choriocapillaris, watershed zones

## Abstract

**Purpose:**

To review the roles of the different vascular beds nourishing the inner retina [retinal ganglion cells (RGCs)] during normal development of the human eye, using our own tissue specimens to support our conclusions.

**Methods:**

An extensive search of the appropriate literature included PubMed, Google scholar, and numerous available textbooks. In addition, choroidal and retinal NADPH-diaphorase stained whole mount preparations were investigated.

**Results:**

The first critical interaction between vascular bed and RGC formation occurs in the sixth to eighth month of gestation leading to a massive reduction of RGCs mainly in the peripheral retina. The first 3 years of age are characterized by an intense growth of the eyeball to near adult size. In the adult eye, the influence of the choroid on inner retinal nutrition was determined by examining the peripheral retinal watershed zones in more detail.

**Conclusion:**

This delicately balanced situation of RGC nutrition is described in the different regions of the eye, and a new graphic presentation is introduced to combine morphological measurements and clinical visual field data.

## Introduction

Non-invasive imaging of the retina has burgeoned in the last decade and opened new possibilities to study the inner layers of the retina and its vascular supply using high magnification *in vivo*. Some of the latest techniques for detecting micro vessels include adapted fluorescence angiography (1, 2), scanning laser ophthalmoscopy (3), and optical coherence tomography (4). In addition, erythrocyte movement can be registered, using a retinal function imager, by calculating “blood flow velocity” and subsequent blood supply over time (5, 6). In the vascularized human retina, the basic functioning hypothesis claims that the inner layers are supplied by capillary branches of the central retinal artery. This is true, for only the central region of the adult retina involved predominantly in acute vision. The developing fetal and adult peripheral retinae have been neglected concerning the source of their nutrition. The need to discuss these topics brought the two authors from different points of view (clinician and scientist) together and formed the basis of this review.

Retinal concepts are often derived from pathological examinations; for the inner retina and optic nerve, common pathological conditions include glaucoma, diabetes, and retinal macular degeneration. Although histological examination might point to underlying physiological dysfunctions, they often focus on specific aspects failing to construct the big picture necessary for a better comprehension. Therefore, one purpose of this work is to review the scientific findings of studies performed on non-pathological conditions (which are partly from the last century) and broaden our understanding of the nutrition and vascular supply of the peripheral retina. This is interesting for both the development of the retinal ganglion cells (RGCs), its vascular beds, and the adult neuronal tissue, which requires oxygen and glucose to survive. The complex aspects of aging are not included here and will be presented in a subsequent publication.

To the best of our knowledge, there is no review to date covering the blood supply of the peripheral retina. Recent reviews on retinal vasculature specialized either on morphological subentities [pericytes (7) and endothelial cells (8)] or covered more specific hemodynamic aspects (9).

To integrate our clinical and laboratory findings, the Goldmann visual field map was modified to demonstrate graphically the number and density of the RGCs and the distribution of the retinal vasculature. Our focus is on non-pathological conditions in an attempt to better understand the normal physiological state.

## Materials and Methods

In this paper, we searched for publications relating to RGCs and retinal vessels. The literature search and the manuscript writing were done between July 2015 and January 2016. The databases used were PubMed, Scopus, Web of Science, and Google Scholar using appropriate keywords in various combinations [retina, ganglion cell, human, development, retinal vessels, vasculature, capillaries, blood supply, nutrition, choroid, and choriocapillaris (CC)]. Textbooks and monographs related to this subject were also used in this literature review.

Many publications related to various keyword combinations were discarded if they (a) did not contain human results, (b) described only pathological situations, or (c) did not contain original research. The time period chosen for the literature search was not restricted and non-English publications were also included.

Using data from the literature, the Goldmann visual field overlay map was modified and superimposed with morphological aspects of the retinal and choroidal vasculature. The graphic modeling was performed manually and by computer. To verify metric numbers given in the literature, our own measurements were performed using tissue samples from previous studies (see below). Because of the small number of these specimens, we did not perform statistics but restricted their use to graphic illustration.

Retinal and choroidal whole mounts, stained with the NADPH diaphorase technique, were reevaluated from a previous publication (10). No new staining or restaining procedures were performed. Unfortunately, only two of the retinae (from donors aged 37 and 40 years) were stained at that time, since the primary focus was on the choroid; the other retinae were used in different protocols or discharged due to long postmortem times. Additional NADPH diaphorase retinal whole mounts were available from the left eye of a previously not described 45-year-old male with no known ocular diseases. The latter was the only set of whole mounts covering all four quadrants up to the ora serrata. The other retinal whole mounts did not include either the far periphery or all quadrants.

The staining procedure of the retinae was parallel to that of the choroidal whole mounts, as described previously (10): the tissue was fixed in neutral buffered 4% paraformaldehyde for 3.5–4 h and then rinsed in phosphate-buffered saline (PBS; pH 7.4) for several times. The retinal whole mounts were freed from the vitreous and the single quadrants incubated free-floating in a staining solution containing of 0.1 mg/ml nitroblue tetrazolium, 1 mg/ml β-NADPH, and 0.3% Triton X-100 in 0.01M PBS. After 2 h, the reaction was stopped in PBS, and the whole mounts were mounted on glass slides (inner retina toward the cover slips) and covered with Kayser’s glycerin jelly.

Measurements and analysis of the whole mounts were performed on different magnified micrographs recorded with a Digital Sight DS-Fi1 camera connected to an Optiphot-2 light microscope (Nikon™).

### Fetal Development of Retinal Blood Supply (Literature Studies)

#### Early General Considerations

Nutrition of the RGCs is a critical factor throughout life. Simple diffusion is limited to the early stages of the embryo and together with the formation of the third germ layer; a specialized vascular system develops specifically in each region of the developing fetus.

The first vessels appearing in the human eye are the hyaloid vessels supplying the lens. The earliest cells to differentiate in the fetal retina are the Müller cells during the sixth week of gestation (wog) (11). Müller cells orient themselves to form radiations centered on the developing optic nerve. They sprout processes, which elongate creating a vertical scaffolding in the retina terminating in the developing internal and external limiting membrane. Their nourishment is derived from the hyaloid vasculature of the primary vitreous.

#### Organization of the Retinal Ganglion Cell Layer

By the seventh (six to eight) wog the RGCs begin to differentiate and migrate from the inner neuroblastic layer along the vertical scaffolding of the Müller’s processes toward the ILM where they will form the RGC layer and sprout axons that grow toward the optic nerve. Groups of adjacent RGCs and their axons (fascicles) are separated from neighboring clumps of RGCs by the Müller cell scaffolding which Radius and Anderson refer to as tunnels (12). We prefer the term “vertical silos” since there is no traffic through them as the term “tunnel” might imply. The RGC layer forms first at 14th wog in the future macular region (13), and then proceeding outward from this immature macula toward the optic disk and ora serrata.

With further differentiation of the RGC layer, there is a loss of the early uniform density of RGCs with a greater concentration occurring at the posterior pole. The total RGC population swells to 2.2–2.5 million by the 18th–30th wog and then decreases rapidly to 1.5–1.7 million cells at birth (14). The loss of the RGCs is up to 10 times greater in the retinal periphery than at the ­posterior pole. The RGC axons in the optic nerve increase in number to an estimated peak of 3.7 million at 16th–17th wog and then decline to 1.1 million at about 29th wog (15, 16). In the retina, optic nerve head, and the optic nerve, there is a scattered arrangement of the peripheral and central axon bundles (fascicles) with a “rough topographic representation” of central and peripheral fiber bundles within the nerve head according to the quadrant they originated in (17). Approximately 70% of the axons in the optic nerve are lost during fetal life.

#### Primary Retinal Ganglion Cell Blood Supply from the Choroid

The RGCs receive their initial nourishment from the primary hyaloid vasculature (vasa hyaloidea propria) but with the involution of the primary vitreous from the developing CC. Between the third and the fourth month of gestation, the main perfusion of the retina is from the CC. Bruch’s membrane appears with its two collagen layers by the 10th–12th wog (11, 18), whereas fenestrations and pericytes in the CC start late in the 22nd wog (19).

#### Capillarization of the Retina

The superficial retinal capillary bed (SCB) begins to form at fourth month of gestation at the base of the hyaloid artery on the optic disk (20, 21). Crucial factors for this step include ligand–receptor systems such as the CXCL12/CXCR4/CXCR7 system described in the developing brain (22, 23) and retina (24). During the sixth month of gestation, the deep retinal capillary bed (DCB) evolves from the SCB (25–28). Similar to other primates, the human DCB consists of two distinguishable layers at the inner and outer margin of the inner nuclear layer (INL) (29). The factors limiting the DCB development to the central part of the retina remain to be determined. One recently identified factor is the low-density lipoprotein receptor-related protein 5 (28); an inhibiting splicing variant of the vascular endothelial growth factor might also play a role (30).

At birth, the SCB has extended nasally to the ora but temporally only beyond the equator (21). The DCB extends temporally just beyond the macula. Consequently, the RGCs in the RGCL located between the fovea and the temporal equator receive their nourishment from the SCB and the outer retina from the CC. Beyond the temporal equator to the ora serrata, the full thickness of the retina, including the RGC layer, receives its nourishment from the CC.

#### Critical Time Periods during Fetal Development

**Figure 1:**
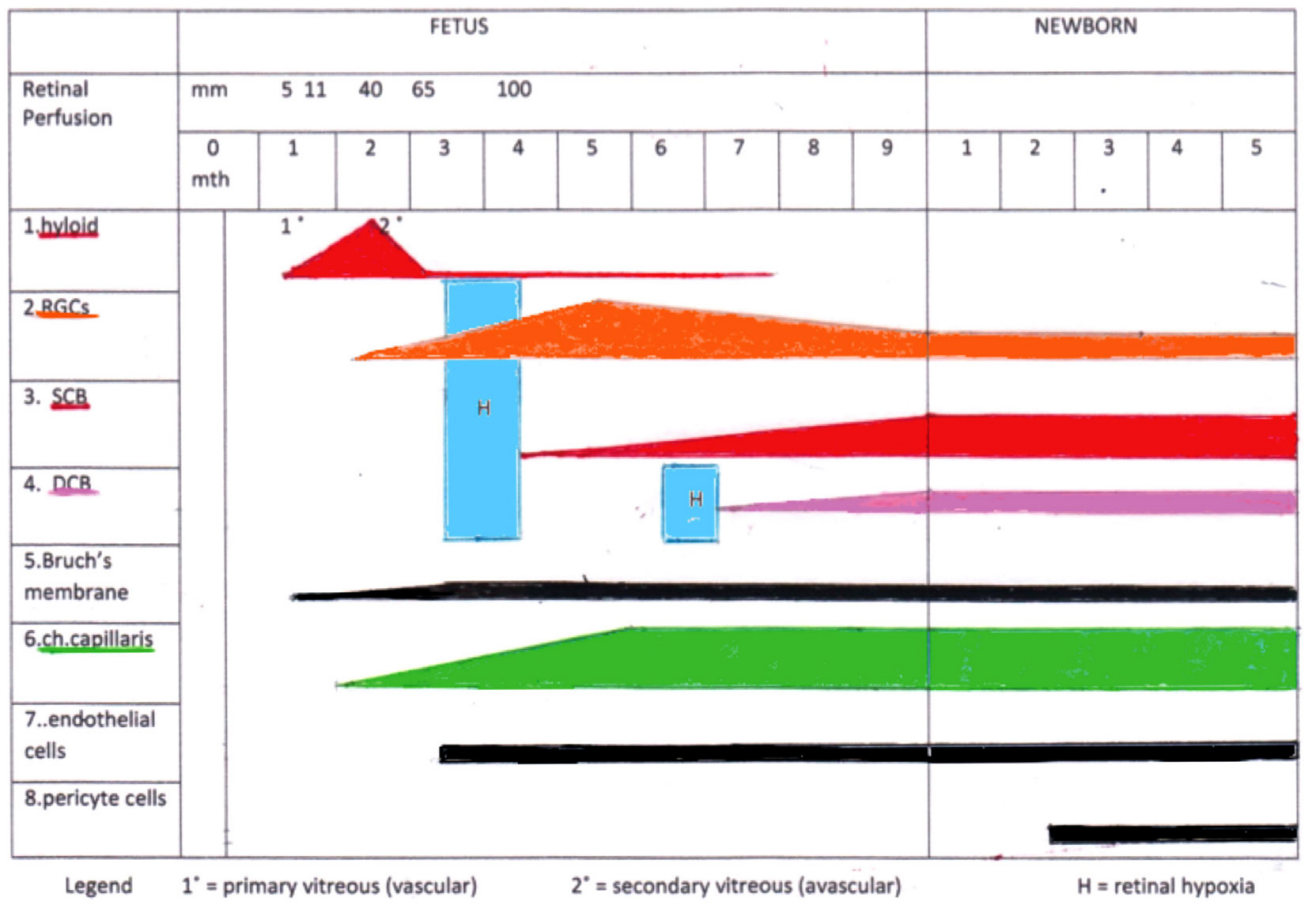
**Summary of the time events during fetal and early newborn stages: formation of the retinal ganglion cells (RGCs), the different vascular beds [hyaloid vessels, superficial retinal capillary bed (SCB), deep retinal capillary bed (DCB), and choriocapillaris (ch.capillaris)], differentiation of the vessels (endothelial cells and pericytes), and Bruch’s membrane**. Note the two critical time frames of potential retinal hypoxia (H) during this development.

The illustration of the vascular and RGC development (Figure 1) demonstrates the critical time periods: the first period occurs at the beginning of the RGCs development when the hyaloid vessels involute and no retinal vessels exist. During this period, the CC develops and supplies the retina exclusively. The second period for critical RGCs nutrition appears right before formation of the DCB in the retina. Due to a general lengthening and thickening of the retina, the choroidal supply becomes insufficient. Consequently, there is a massive apoptotic degeneration of RGCs. This effect is greatest in the peripheral retina where no DCB exists.

### Early Childhood Changes (Literature Studies)

After birth, the temporal SCB grows from beyond the equator to 1–2 mm short of the ora serrata by the fifth month of life (21, 29). This 1–2 mm of full thickness retina posterior to the ora serrata is perfused by the underlying CC.

Pericytes in the capillary walls begin to appear by the second month after birth and are present in all the capillaries by the third to fifth month except in the extreme retinal periphery (21). Both retinal capillary beds assume their adult configuration with capillary meshwork of varying sizes and shapes by the fifth month after birth.

The fetal eyeball grows rapidly and reaches a sagittal diameter of 12–14 mm at birth. There is continued rapid growth until 3 years of age, when the sagittal diameter reaches 23 mm. Over the next 10 years, it increases only 1–24 mm (31). In essence, the eyeball enlarges 50% after birth. Using this figure of 50% with Michaelson’s adult measurements (20) of the extent of the SCB and the DCB, one can determine their location at birth. The exception is the temporal extent of the SCB, which only reaches the ora 5 months after birth.

By the age of 13 years, the temporal retina attains its adult dimensions with the temporal macula enlarging 1.39 mm, the equator 6.45 mm, and the very peripheral retina posterior to the ora 1.61 mm (31). It is the equatorial region of the newborn that increases the most in comparison to the ora and macular region. The increase in the adult size of the capillary nets of the SCB in the equatorial region when compared to that of the macular region can be explained by both the postpartum growth of this region and by the loss of peripheral RGCs during the second trimester (15, 16). The apoptosis of 700,000 RGCs could lead to collapsing of many vertical silo complexes and a resultant increase in the size of the surrounding peripheral complexes.

### Adult Morphology (Literature Studies)

#### Retinal Ganglion Cells

Using different techniques to estimate the number and density of RGCs in the retina (32–34) and their axons in the optic nerve (35), it was found that their number and density in donors up to 50 years of age was quite stable (all studies were cross-sectional designs providing data to one single time point only; the sample size varied between three and seven donors; donor age ranged between 16 and 48 years). It is interesting to note that most later published estimations of RGCs in the human retina are based on these data (36, 37) or using data from non-human primates (38).

Vrabec measured the diameter of the RGC axons bundles (fascicles) in the temporal macular region and found them to be ~60 μm in diameter (39). These axon bundles in the adult continue to be isolated from their neighboring axon bundles by the sheaths created by Müller cell processes during fetal development (12).

#### General Retinal Vascular Supply

Each of the four quadrants of the retina centered on the optic nerve is perfused by a single retinal artery (end artery) whose SCB ends 1–2 mm behind the ora serrata. Each retinal artery is drained by a single retinal vein with infrequent arteriovenous anastomoses or direct communications with the underlying choroid or the peripheral ciliary body blood vessels. The superior hemiretina is separated from the inferior hemiretina by a horizontal raphe present in the nasal and temporal retina. The temporal horizontal raphe excludes the macula and the papillomacular bundle (PMB). The horizontal raphe crosses the equator ~18.5 mm nasally from the CRA on the optic disk and 22.5 mm from the optic disk temporally (40). The posterior portion of the choroid circulation is perfused by the short posterior ciliary arteries surrounding the optic nerve. The anterior portion of the choroid receives the blood supply from recurrent branches of the long posterior ciliary arteries and the anterior ciliary artery (41). In the retina, the concept of watershed zone exists for both vascular perfusion and tissue diffusion. The peripheral retina has two vascular watershed zones effecting its perfusion: one, along the temporal horizontal raphe, and the other, at the ora serrata.

#### Retinal Capillary Beds

Functional considerations allow the subdivision of retinal vessels into three capillary beds.

The SCB traverses the RGC layer at varying levels within this layer and provides nourishment for both the RGCs and the RGC axons in the retinal nerve fiber layer (42). The diameter of the average capillary meshwork width of the SCB at the posterior pole is 65 μm, which is similar to the 60 μm diameter of the RGC axon bundles, which they nourish (20, 39). The SCB covers most of the retina leaving only the fovea centralis and the most peripheral retina next to the ora serrata avascular.

The DCB lies in the INL encompassing the posterior pole where it anastomoses freely with the SCB. From the center of the optic disk, it extends 14.5 mm in the lateral horizontal axis (20, 43). This is equivalent to 33.3° temporally to fixation on the retinal overlay map. Consequently, the conversion of the other three quadrants from the literature equals nasal 41.6°, superior 35.6°, and inferior 38.3°. The DCB is only present when the thickness of the retina exceeds 100 μm, when the distance of the SCB to the outer nuclei of the INL is more than 45 μm (33, 44). Diffusion of nutrients is the final link in the nutrition chain to the RGCs. Iwasaki and Inomata (42) estimated that the maximal diffusion distance of the capillary net in the SCB is 45 μm. Thus, diffusion is optimal in capillary nets <90 μm in diameter.

First described by Michaelson and Campell (45), the retinal *peripapillary* capillary bed supplies the inner aspect of the NFL extending from both the superior and inferior poles of the optic disk in an arc along the great vessel arcade to a greater extent temporally than nasally. It arises from the retinal circulation adjacent to the optic disk, which also supplies the capillaries on the surface of the optic nerve head. These surface optic disk capillaries communicate with the capillaries below in the prelaminar region, which arise from the short posterior ciliary arteries. [For a detailed description of the human optic nerve vascular anatomy, see a most recent review by Mackenzie and Cioffi (46).]

#### General Aspects of the Choriocapillaris

The wall of the CC is composed of endothelial cells and few pericytes located in the lateral or outer part of the CC wall. The thickness of the CC measures about 10 μm at the fovea and 7 μm in the periphery, whereas the diameter is between 20 and 50 μm (47). It has fenestrations in its walls facing Bruch’s membrane that allow the passage of macromolecules. The architecture of the capillary bed changes at the equator from a lobular organization at the posterior pole to ladder-like organization extending to the ora (18).

Exhibiting a slow blood velocity (48), it supplies the retinal pigment epithelium (RPE) and outer retina with oxygen and nutrients and removes the waste products of both the RPE and outer retina. Wybar estimated that the retina could be nourished for a distance of 120–140 μm by the underlying CC (43).

### Adult Morphology (Own Observations)

#### Projection of Morphological Features on a Goldmann Visual Field Template

**Figure 2:**
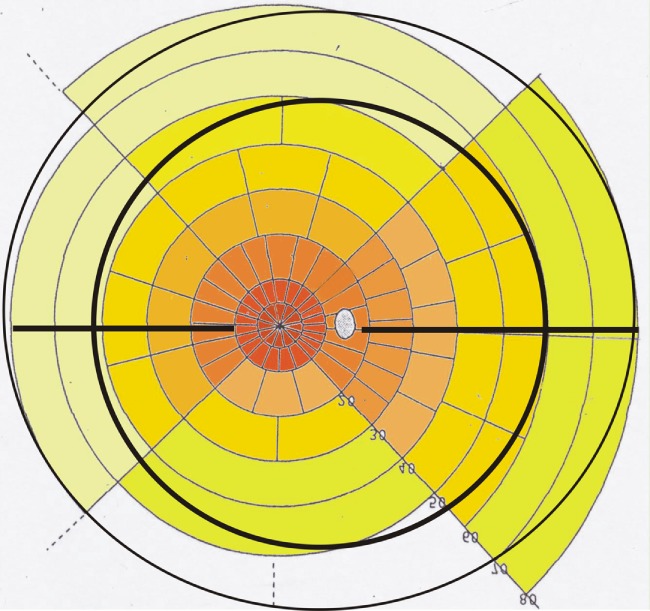
**Retinal ganglion cell overlay map of the right eye: note the projection of the ora serrata (outer black circular line), the horizontal raphe (black horizontal line), and the equator (inner black circular line) on a modified Goldmann visual field map using data from Ref. (32, 44)**. The density of ganglion cells is color coded from red–brown (high density) to light yellow (low density). Left side is temporal and right side is nasal retina. Each field marks the area of 1% of the retinal ganglion cells. The distance from the fovea region (white center) is 5° for the first circle, 10° for the second, 20° for the third, and continuing in 10° steps. The dotted white area marks the optic nerve.

**Figure 3:**
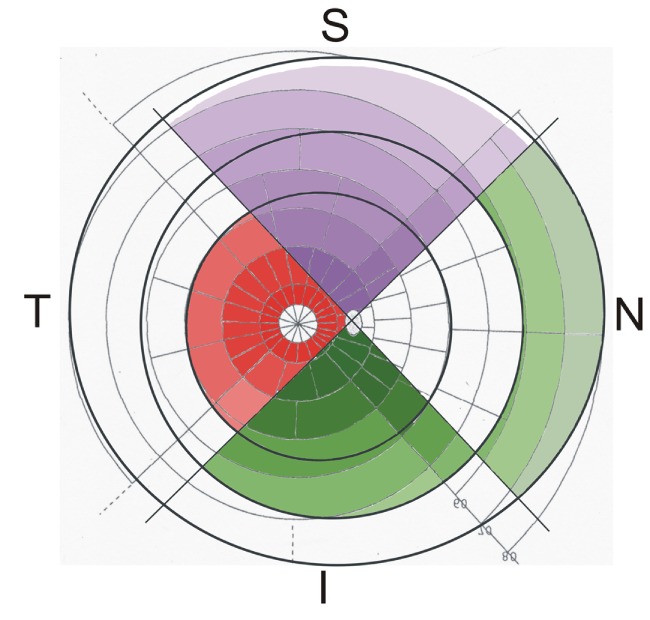
**Watershed zones of the retina and choroid, projected on a modified Goldmann visual field map, the color saturation representing the density of the retinal ganglion cells (less saturation means less density): in the superior quadrant (S), the superficial capillary bed of the retinal vessels (purple) covers most of the retina leaving only a small free area next to the ora serrata (outer dark black ring)**. The deep capillary bed of the retinal vessels (marked red in the temporal quadrant T) is only present in the central part of the retina (edge marked by the inner dark black ring). The posterior choriocapillaris (CC; marked in the inferior quadrant I) reaches to the equator (middle dark black ring) and is continuous with the anterior CC (marked in the nasal quadrant N). Note that the peripheral retina supplied solely by the superficial retinal capillary bed has an inner part supported by the posterior CC and an outer part supported by the anterior CC.

To better understand the functional topography of retinal RGC density in the healthy adult retina, we used as a template the Goldmann visual field overlay in a unique manner. This template created by Rizzo and Castelbuono was based on the human RGC studies performed by Curcio (32). By reversing the template along the *X* and *Y* axes, the RGC overlay becomes a map of the location of the RGC density of the normal retina of the right eye. Each sector represents 1% of total RGCs of retina; 10° of the visual field corresponds to 3 mm on the retinal surface (44, 49). Projection of the morphological measurements concerning the ora serrata and the equator of the eyeball to this template are shown in Figure 2. The different reference points (clinical visual field measurements refer to the fovea, morphological measurements refer mainly to the optic nerve) often confuse the combination of these data sets. In the graphic presentation presented, one can easily read that the equator projects temporal only to the 40° line (in contrast to the 60° line nasally) while the distance from the optic nerve is slightly longer toward the temporal side. The morphological data become especially interesting when projecting the different vascular beds on the visual field map (Figure 3).

In this figure, the density of the RGCs is not only represented by the sectors but also by differences in the color saturation (high saturation means high density of ganglion cells). Concerning the peripheral retina, which is supported only by the SCB of the retinal vessels, two different parts can be distinguished: an inner part, reaching from the end of the central retina toward the equator is characterized by a normal architecture of the CC (posterior CC, presented in the inferior quadrant of Figure 3), and an outer part, covering the most peripheral region of the retina anterior of the equator (anterior CC, presented in the nasal quadrant of Figure 3). The outer part is especially vulnerable, since it reaches the watershed zone next to the ora serrata and receives a different choroidal blood supply.

#### Studies on Retinal Whole Mounts

Measurements on NADPH-diaphorase stained retinal whole mounts confirmed the estimated temporal and nasal extension of the DCB (9.6–10.6 mm temporal, equals 32°–35°; 12.6–13.5 mm nasal, equals 42°–45°) but showed a smaller extension in the superior and inferior quadrants (9.0–9.6 mm superior, equals 30–32°; 9.0–9.9 mm inferior, equals 30°–33°).

**Figure 4:**
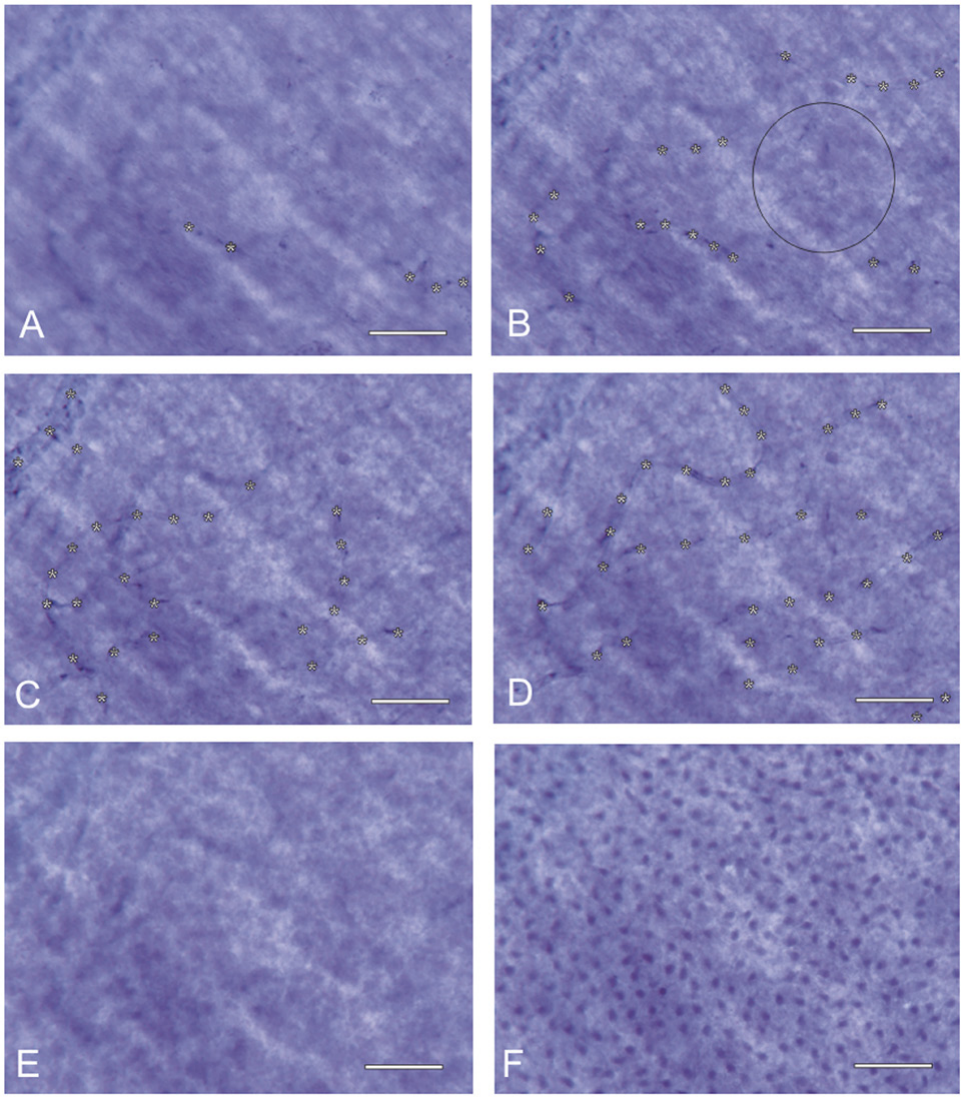
**Inner retinal nutrition of the central retina: different foci of a NADPH-diaphorase stained retinal whole mount (~20° temporally; donor 45 years of age) show the overlay of the superficial and deep capillary bed (capillaries in focus marked by asterisks)**. The distance of all retinal regions to the nearest capillary is always <45 μm: the circle in **(B)** has a diameter of 90 μm. Even if it does not touch a vessel in this sectional plane, it contacts vessels shown in **(D)**. **(A)** nerve fiber layer, **(B)** ganglion cell layer, **(C)** inner inner nuclear layer, **(D)** outer inner nuclear layer, **(E)** outer nuclear layer, and **(F)** photoreceptor layer. Scale bar = 50 μm.

In the *central region* of the retina, the proper retinal capillary density is secured by the DCB, which projects between the SCB loops. When zooming through different layers of the central retina (Figure 4), it becomes apparent that those areas, which show intercapillary distances of the SCB of more than 75 μm (vessels marked in Figure 4B), show capillaries of the DCB (vessels marked in Figure 4D) within these areas (theoretical overlay of Figures 4B,D). The central RGCs are therefore completely supplied by the retinal vessels without participation of the choroid.

**Figure 5:**
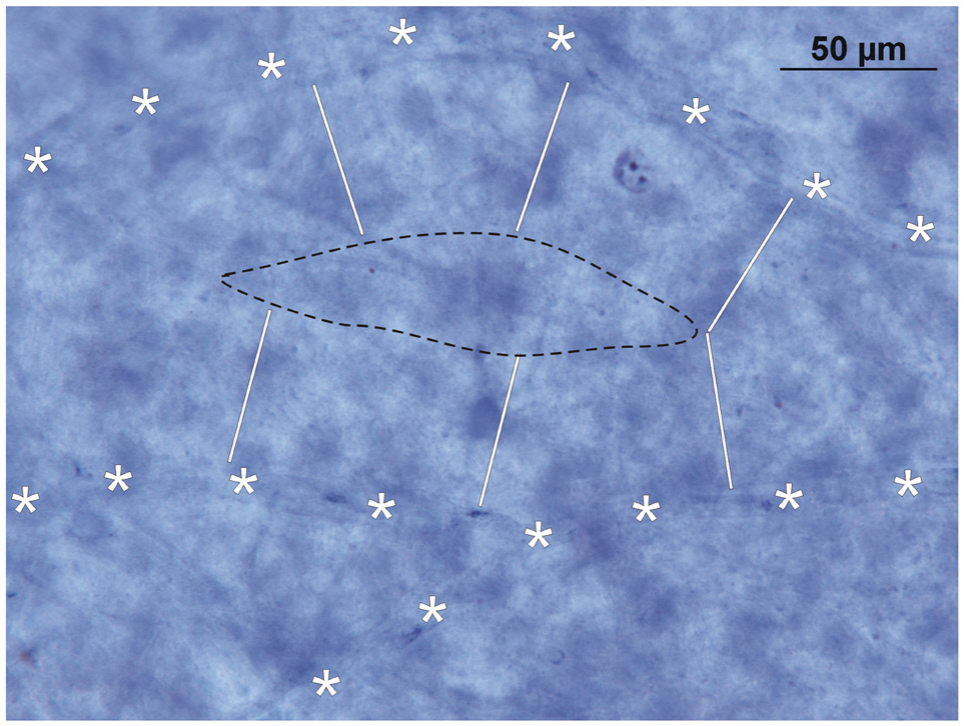
**Inner retinal nutrition of the peripheral retina: NADPH-diaphorase stained retinal whole mount (~50° temporally; donor 45 years of age)**. Note that due to the diffusion limit of the superficial capillary bed (white bars from the retinal capillaries, marked with asterisks, show the critical distance of 45 μm) only part of the inner peripheral retina is sufficiently supplied by oxygen and nutrition. The area within the black broken line has to be supplied by the choroid.

In the fetal *peripheral retina*, the generation of Müller’s vertical silos shows little, if any, difference to those of the central retina. Because of the loss of RGCs at the end of the fetal period and along with the intense growth of the eye during the first 3 years of life, the peripheral SCB shows diameters larger than 90 μm between single capillary loops. In Figure 5, the diffusion distance from neighboring capillaries (marked by asterisks) is marked by white bars. As a consequence, the retinal vascular supply does not reach the center (marked by a black broken line in Figure 5) of the vertical silos. Since there exists no DCB, sufficient nutrition of RGCs in these areas is only possible by the underlying CC. Therefore, the peripheral RGCs depend on both the retinal and choroidal vascular supply and are thus more vulnerable to changes in either of their vascular beds.

## Conclusion

Reviewing the published literature showed that during normal fetal development the inner layers of the human retina (mainly the ganglion cell layer) change their vascular supply from a vitreal to a choroidal and then a mixture of both retinal and choroidal blood vessels. This change is not equal for all regions of the retina, because the RGCs of the central retina received their nutrition from the SCB and the DCB, but the peripheral RGCs remain dependent for their nutrition on the CC and the SCB. Most clinical visual field investigations (e.g., the 24-2 Humphrey visual field threshold test) focus on the central retina; consequently, they fail to record information from the peripheral retina and its interacting with the underlying choroid. This information is crucial for a better understanding of inner retina aging. Virtually, little data exist at present about changes of the peripheral inner retina concerning age or diseases affecting the inner retina (such as glaucoma). It is tempting to speculate that with more comprehensive peripheral retinal examinations (clinically and morphologically), one might better understand the early changes occurring in the peripheral ganglion cell layer, which appears to be more vulnerable, because of its critical dependence for nutrition on both the retinal and choroidal capillaries. New techniques are on its way to include the more peripheral regions, e.g., the 60-4 Humphrey visual field test (50), the 60° OCT (51), and the ultra wide field camera (52). In this regard, changes of the choroid might be more important than previously thought.

## Author Contributions

PR initiated the study, had the ideas of the new graphic presentation, and wrote the manuscript. C-AM made profound research, added the original micrographs, and wrote the manuscript.

## Conflict of Interest Statement

The authors declare that the research was conducted in the absence of any commercial or financial relationships that could be construed as a potential conflict of interest.
